# First principles modeling of pure black phosphorus devices under pressure

**DOI:** 10.3762/bjnano.10.190

**Published:** 2019-09-24

**Authors:** Ximing Rong, Zhizhou Yu, Zewen Wu, Junjun Li, Bin Wang, Yin Wang

**Affiliations:** 1 Key Laboratory of Optoelectronic Devices and Systems of Ministry of Education and Guangdong Province, Shenzhen Key Laboratory of Advanced Thin Films and Applications, College of Physics and Optoelectronic Engineering, Shenzhen University, Shenzhen, 518060, China; 2 Department of Physics and International Centre for Quantum and Molecular Structures, Shanghai University, 99 Shangda Road, Shanghai 200444, China; 3 Center for Quantum Transport and Thermal Energy Science, School of Physics and Technology, Nanjing Normal University, Nanjing 210023, China; 4 Department of Physics and Shenzhen Institute of Research and Innovation, the University of Hong Kong, Pokfulam Road, Hong Kong SAR, China; 5 Hongzhiwei Technology (Shanghai) Co., Ltd. Shanghai 200000, China; 6 Center for Quantum Computing, Peng Cheng Laboratory, Shenzhen 518060, China

**Keywords:** band alignment, black phosphorus, first principles calculation, pressure sensors, WKB approximation

## Abstract

Black phosphorus (BP) has a pressure-dependent bandgap width and shows the potential for applications as a low-dimensional pressure sensor. We built two kinds of pure BP devices with zigzag or armchair conformation, and explored their pressure-dependent conductance in detail by using first principles calculations. The zigzag BP devices and the armchair BP devices exhibit different conductance–pressure relationships. For the zigzag BP devices conductance is robust against stress when the out-of-plane pressure ratio is less than 15%, and then increases rapidly until the conductive channels are fully opened. For the armchair pure BP devices conductance decreases at first by six orders of magnitude under increasing pressure and then increases quickly with further increase of pressure until the devices enter the on-state. This shows that the pure zigzag BP devices are more suitable for the application as flexible electronic devices with almost constant conductance under small pressure, while armchair BP devices can serve as bidirectional pressure sensors. Real-space distributions of band alignments were explored to understand the different pressure-related properties. We fitted a set of parameters based on the results from the empirical Wentzel–Kramers–Brillouin method, which provides an effortless approximation to quantitatively predict the pressure-related behaviors of large pure BP devices.

## Introduction

Black phosphorus (BP) has been regarded as one of the most popular two-dimensional (2D) materials due to their unique properties and potential applications in many fields of nanoelectronics [1–3]. So far, many studies have been carried out to explore the electronic properties [3–6], optical spectra [7–10], excitons [11–13], quantum transport [14–18], plasmons [5,19], thermoelectric effects [20–21], and superconductivity [22–24] of BP. One of the most promising applications of BP at the industrial level is expected to be the use as field-effect transistor [1,25–28]. Different from the planar 2D materials, such as graphene and silicene, the puckered configuration of BP makes structural deformation much easier by tension or compression along any direction. Meanwhile, large-scale bandgap modulation accompanied by high carrier mobilities can be realized, which are two main focuses of nanoscale electronic devices [1,3,7,13,25,29–31]. Previous studies indicated that BP can withstand a tensile stress as high as 10 N/m and a strain up to 30%, and exhibit a direct–indirect–direct bandgap phase transition under axial strain [5,32]. The sensitivity to and the resilience against strain make BP an ideal material for strain-sensing electronics and flexible electronic devices. Xiao et al. fabricated few-layer BP nanosheets by chemical vapor transport [25], and observed a phase transition from an orthorhombic semiconductor to a simple cubic metal with increasing pressure by performing in situ ADXRD and Raman spectroscopy with the assistance of a DAC apparatus. They also carried out first principles calculations to interpret the metallic behavior of BP under pressure. Pablo et al. investigated the funnel effect in monolayer BP [13], which describes the possibility of controlling exciton motion by means of inhomogeneous strains. They found that the funnel effect in BP is much stronger than that in MoS_2_, and more important, shows opposite behavior to that in MoS_2_. Excitons in BP are mainly accumulated isotropically in strain-reduced regions, instead of occurring in the regions with a high tensile strain like in MoS_2_. Deniz et al. investigated the strain-related optical properties of monolayer BP using first principles calculations [7]. They found that the optical response of BP is sensitive to the magnitude and the orientation of the applied strain due to the strong anisotropic atomic structure of BP. Based on first principles calculations, Koda et al. studied the electric behavior of BP-MoSe_2_ and BP-WSe_2_ heterobilayers, and analyzed the effect of long-range structural bending on the electronic properties through van der Waals interactions [29]. Although the pressure response of BP has been extensively studied, the dependence of strain-related quantum transport on the conformation in pure BP devices was rarely explored. This is the purpose of this manuscript. Moreover, in addition to study the chirality dependence of strain-related quantum transport of BP, we propose an empirical model based on the Wentzel–Kramers–Brillouin (WKB) approximation to describe the transport through the BP device, which could serve as a simple compact model for this emerging device.

In this manuscript, we built a set of pure monolayer zigzag and armchair BP nanodevices and investigated their pressure-related electric properties from first principles. Comparing to metal–BP heterojunctions, pure BP devices have advantages including simple structure, easy preparation, smooth continuous interface, and avoidable lattice mismatch. We want to address the following questions of pure BP devices. 1) How does the conformation of pure BP devices influence the pressure-dependent quantum transport? Is either the zigzag or the armchair device suitable for pressure sensor applications? 2) How does the magnitude of pressure influence the transport properties of the pure BP devices? 3) How does their length influence the conductance of pure BP devices? 4) Can the conclusions from the system of finite size be transferred to larger scales? To answer these questions, first principles calculations were carried out to investigate the quantum transport of pure BP devices within the framework of combination of non-equilibrium Green’s function (NEGF) and density functional theory (DFT) [33–34].

The manuscript is organized as follows: In section “Simulation Details”, we show the pure zigzag and armchair BP nanodevices and introduce the first principles modeling methods. In section “Results and Discussion”, we present the numerical results and physical analysis of the BP devices including pressure-dependent mechanical and electric properties, quantum transport and band alignment. In addition, the conductance of the BP devices obtained from the classical WKB approximation is provided. Finally, a conclusion of this manuscript is given.

## Simulation Details

**Figure 1:**
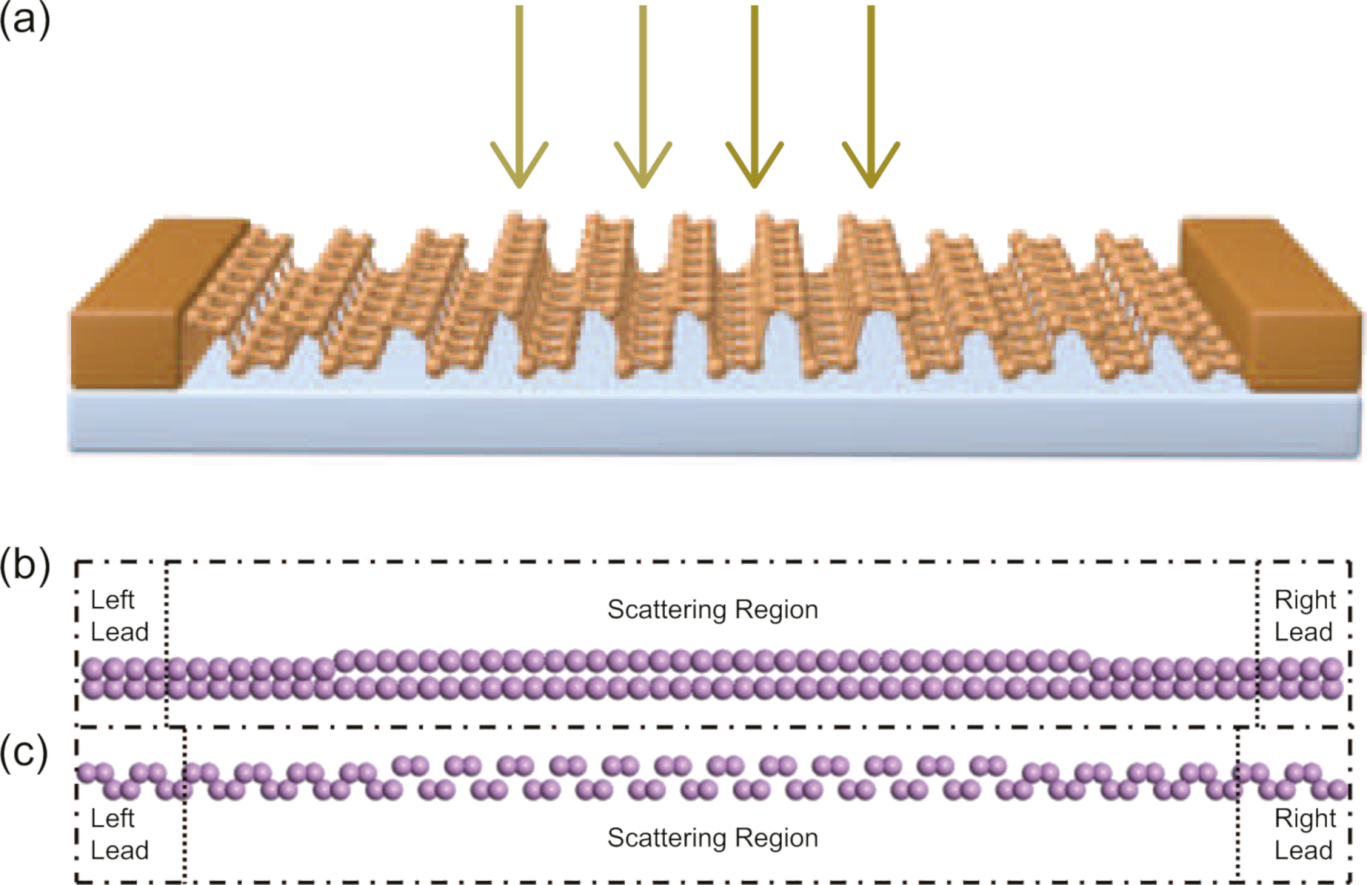
(a) Schematic drawing of the BP nanodevice; (b) zigzag BP device with a length of the central region of 18*L*; (c) armchair BP device with a length of the central region of 12*L*. Here, *L* represents the length of the unit cell of BP along zigzag direction or armchair direction.

Figure 1a shows a schematic drawing of the proposed BP nanodevices, and Figure 1b and Figure 1c show the atomic structures of the two different pure BP nanodevices, in which quantum transport is along the zigzag direction or the armchair direction of BP, respectively. For each device, two probes are formed by compressing the BP with a fixed pressure ratio of 30%, and the central region is composed of a section of BP with tunable pressure ratio from zero to 30%. In this investigation, the pressure ratio is defined as *R*_C_ = (1 − *h*/*h*_0_) × 100%, where *h*_0_ and *h* represent the thickness of free-standing and compressed BP along the direction vertical to the BP plane, respectively. The length of the central region was chosen equal to 4*L*, 6*L*, 12*L*, 18*L*, and 24*L*, where *L* ca. 3.33 Å for the zigzag BP device and ca. 4.63 Å for the armchair BP device indicating the length of a unit cell of BP along the zigzag and armchair directions, respectively. Both zigzag and armchair BP devices are periodic in the direction perpendicular to the quantum transport in the BP plane. We want to emphasize that the size of the largest structures with a pressure region of 24*L* is already compatible with the practical scale of a transistor.

In our investigation, two different DFT codes, VASP [35–36] and NanoDCal [33–34], were used to model the electric behavior and quantum transport of the BP devices under pressure, respectively. The structural relaxations of all compressing procedures of BP were implemented by VASP, and accomplished by using the Perdew–Burke–Ernzerhof (PBE) functional along with projector augmented-waved (PAW) potentials [37–38]. The kinetic energy cutoff was chosen to be 500 eV and the reciprocal space was meshed by 13 × 9 × 1 using the Monkhorst–Pack method [39]. The volume of the structures was fully relaxed until the atomic force was smaller than 0.001 eV per angstrom. The relaxed lattice constants for a free standing BP unit cell are 4.625 Å in armchair direction, 3.298 Å in the zigzag direction, and 2.102 Å in the vertical direction between two non-equivalent P atomic layers, which is in good agreement with the recognized DFT results [32].

The quantum transport properties of BP devices were implemented by the transport package NanoDCal, which is based on the standard NEGF-DFT method [33–34]. In our calculation, norm-conserving non-local pseudo-potentials were used to define the atomic cores [40], and an atomic orbital basis set with single-ζ plus polarization was used to expand physical quantities [41]. The exchange correlation potential was treated using the PBE functional [37–38]. Finally, the NEGF-DFT self-consistency was carried out until the numerical tolerance of the Hamiltonian matrix was below 10^−4^ eV.

## Results and Discussion

### Mechanical properties of BP under pressure

In this section, we discuss the mechanical behavior of periodic 2D BP with the increase of pressure along the direction vertical to the 2D plane. Normally, a vertical pressure causes the expansion of BP in both zigzag and armchair directions, although the deformation along the latter is much easier than that along the former because the Young’s modulus in the armchair direction (44 GPa) is much smaller than that in the zigzag direction (166 GPa) [5]. However, we ignored the pressure-induced expansion in this investigation, and only relaxed the relative locations of P atoms inside a fixed-size box for a given value of *R*_C_ to obtain partially relaxed structures. This is reasonable considering the restriction of 2D quantum devices where the size of scattering region must be fixed to ensure the periodic extension along the transverse direction. In addition, we also did some calculation for fully relaxed structures as a comparison, where monolayer BP is relaxed at different *R*_C_ inside a box with tunable size.

**Figure 2:**
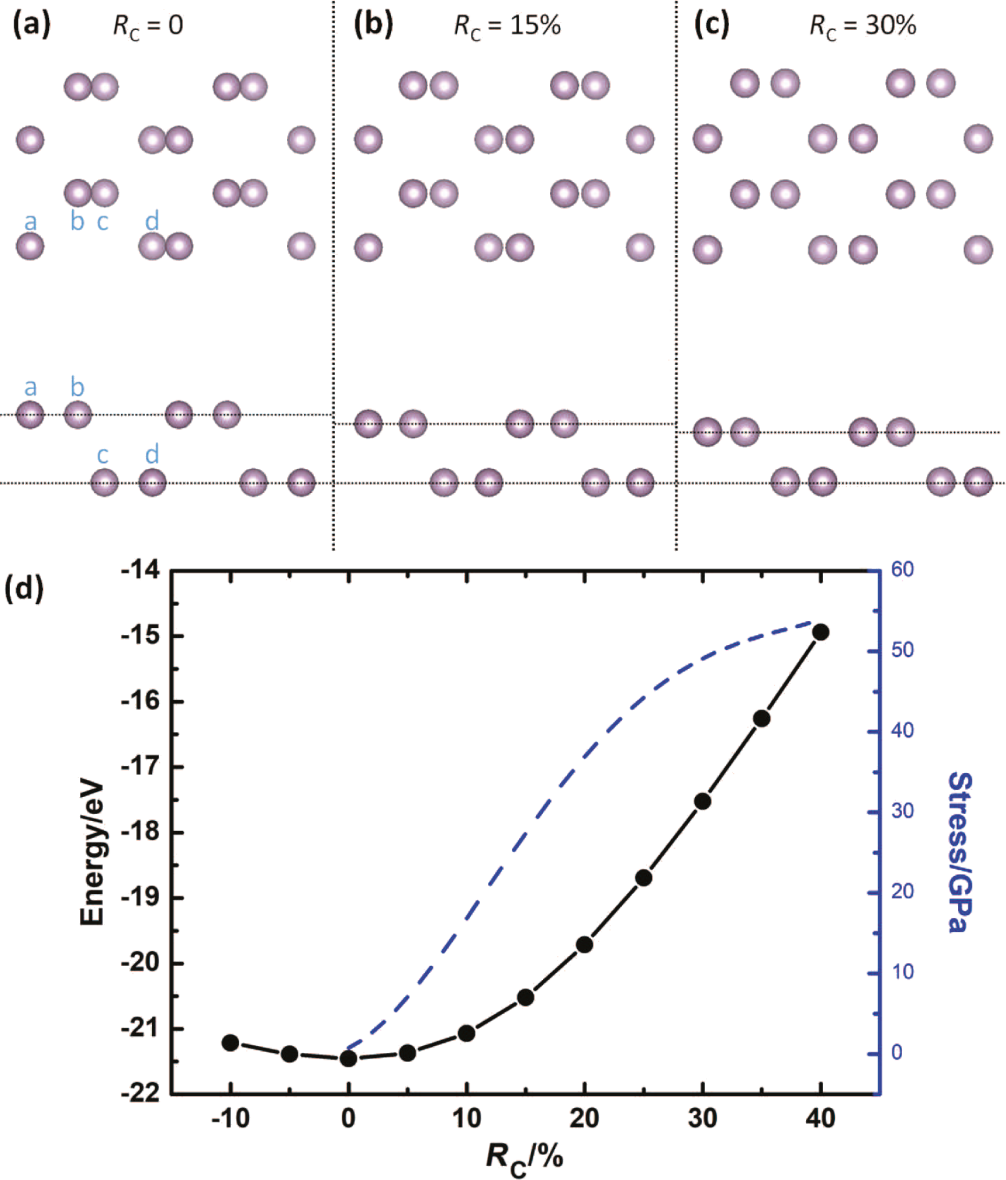
(a–c) Top view and side view of the partially relaxed BP structures under different compressing ratios *R*_C_. (d) Total energy *E*_tot_ (black solid circles) and stress *P*_s_ (blue dashed curve) of a monolayer BP unit cell as functions of the pressure ratio *R*_C_.

Figure 2a–c presents top view and side view of a monolayer BP under pressure with *R*_C_ = 0%, 15%, and 30%, respectively, where “a”, “b”, “c”, and “d” represent the P atoms in the unit cell. With the increase of *R*_C_ from zero to 30%, the distance between atom “a” and atom “b‘” (or atom “c” and atom “d”) in the same layer decreases from 1.49 Å (*R*_C_ = 0) to 1.11 Å (*R*_C_ = 30%), while the distance between atom “b” and atom “c” increases from 0.83 Å to 1.2 Å. The distance between atom “a” and atom “b” (or atom “c” and atom “d”) along the zigzag direction remains constant at 1.65 Å.

In order to estimate the mechanical behavior of a monolayer BP under pressure, we calculated the total energy *E*_tot_ and stress *P*_s_ at a unit cell as functions of pressure ratio *R*_C_. As shown in Figure 2d, *E*_tot_ decreases when *R*_C_ increases from −10% to zero, and then rises up smoothly from −21.4 eV to −14.8 eV with *R*_C_ changing from zero to 40%, which indicates the stability of monolayer BP under zero pressure. The smooth variation of *E*_tot_ means that monolayer BP can maintain its structure even at large pressure, and does not form any structural defect or bonding twist. The stress *P*_s_ can be calculated by *P*_s_ = −∂*E*_tot_/∂*d*·*1*/*S* with *d* the thickness of BP and *S* the pressure area [42–44]. As shown in Figure 2d, *P*_s_ increases continuously from zero to 54 GPa when *R*_C_ changes from zero to 40%. This is reasonable because it becomes more and more difficult to bring the atoms closer together with increasing pressure. By fitting the stress curve from *R*_C_ = 0 to *R*_C_ = 5%, the Young’s modulus vertical to the BP plane was obtained with a value equal to 127 GPa.

### Electric properties of BP under pressure

**Figure 3:**
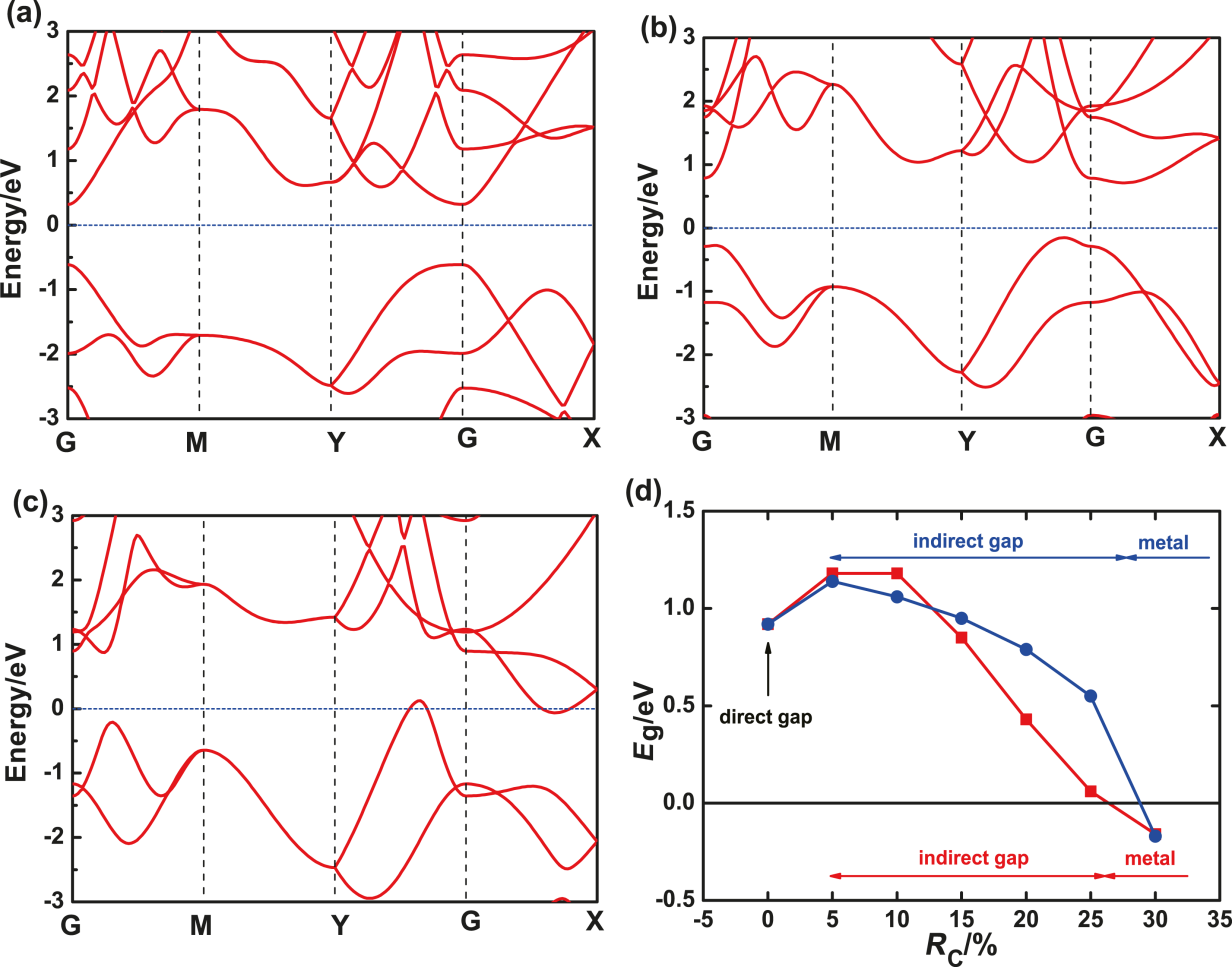
(a–c) Band structures of partially relaxed BP with pressure ratio *R*_C_ = 0, 15% and 30%, respectively. (d) Bandgap energy *E*_g_ as a function of *R*_C_ for partially relaxed (blue circles) and fully relaxed (red squares) 2D BP. The black horizontal line at *E*_g_ = 0 indicates the Fermi level.

In this section, we show the electric behavior of 2D pressure-related monolayer BP. Figure 3a–c shows the band structures of a partially relaxed monolayer BP with *R*_C_ equal to 0,15%, and 30%, respectively. When *R*_C_ = 0, a direct bandgap of ca. 0.91 eV appears at the Γ point, which is in agreement with several theoretical works [12,32]. Although this bandgap is smaller than that measured in experiments [4,11] and obtained from the GW method [12], the substance of pressure-related quantum transport in pure BP devices in this paper is not changed. When *R*_C_ = 15%, monolayer BP becomes an indirect bandgap semiconductor. When *R*_C_ = 30%, the conduction band minimum (CBM) descends below the valence band maximum (VBM), and the monolayer BP finally becomes a conducting material.

Figure 3d shows the bandgap energy of a partially relaxed monolayer BP as a function of *R*_C_ indicated by the blue solid circles. For comparison, the bandgap energy of a fully relaxed BP is also plotted with red solid squares. The bandgap energy increases first and then decreases with increasing *R*_C_. When *R*_C_ reaches ca. 5%, the monolayer BP changes from a direct bandgap semiconductor to an indirect bandgap semiconductor. With further increase of *R*_C_, the bandgap decreases continuously, and the monolayer BP eventually becomes a conductor when *R*_C_ is 25–30%. The fully relaxed BP and partially relaxed BP show qualitatively consistent behaviors of bandgap variation and even the same phase transition points, although the bandgap energy of the fully relaxed BP decreases faster when *R*_C_ increases from 10% to 25%.

### Conductance of pure BP devices under pressure

**Figure 4:**
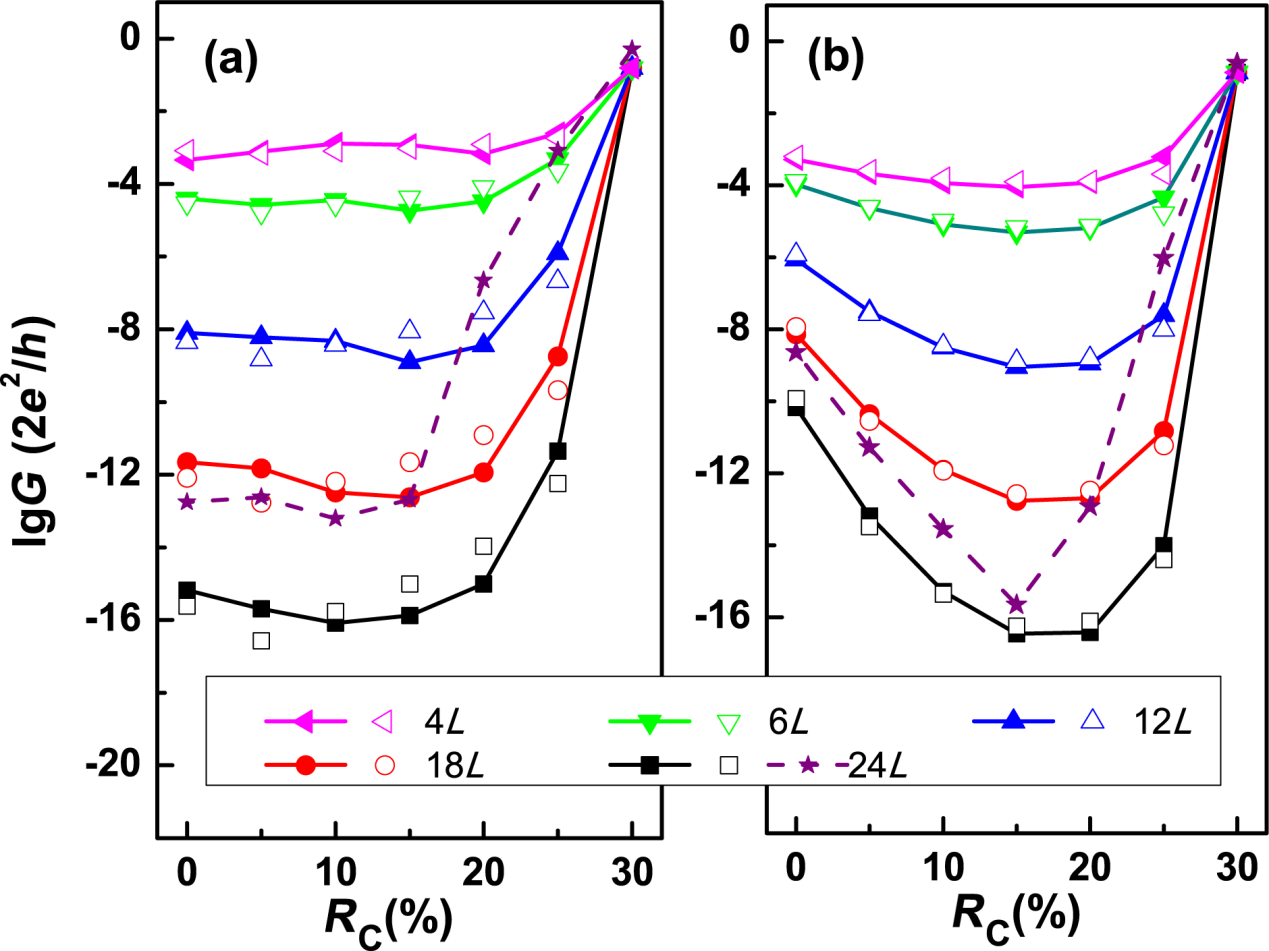
Conductance *G* from first principles calculations (solid symbols) and *G*_WKB_ fitted by the WKB model (open symbols) in logarithmic scale a functions of *R*_C_ for (a) zigzag BP devices, and (b) armchair BP devices with different lengths of the pressure region. In each panel, the dashed curve with stars represents the conductance of a fully relaxed monolayer BP device with a length of the pressure region of 24*L*.

In this subsection, we show the pressure-related conductance of pure monolayer zigzag and armchair BP devices. The transmission coefficient of a two-probe system can be calculated by,

[1] $$T\left(E\right)=Tr\left(\Gamma_{\text{L}}G^{\text{R}}\Gamma_{\text{R}}G^{\text{A}}\right),$$

where *G*^R^ and *G*^A^ are the retarded and advanced non-equilibrium Green’s functions of the system, respectively, and Γ_L_ and Γ_R_ are the line-width functions describing the interaction between leads and scattering region. The conductance *G* at the equilibrium state is defined as *G* = 2*e*^2^/*h*·*T*(*E*_F_) with *E*_F_ the Fermi level. Figure 4a and Figure 4b show the length- and pressure-dependent conductance *G* at equilibrium for the zigzag BP devices and armchair BP devices, respectively. For each device, *R*_C_ is fixed to 30% for both BP leads, while *R*_C_ changes from zero to 30% for the central BP section in the scattering region.

For the zigzag BP devices, several information can be obtained from Figure 4a. First, the conductance *G* decreases as a function of the length of the scattering region when *R*_C_ is less than 30%. This is reasonable because a longer structure corresponds to a wider potential barrier in the scattering region due to the semiconducting BP section in the central region. Secondly, the variation of *G* is very small for each structure when *R*_C_ is smaller than 15%, which indicates a possible application of zigzag BP devices as flexible electronic circuits [14,16,45]. Thirdly, *G* increases dramatically at *R*_C_ *>* 20% and finally reaches a comon value for all zigzag BP devices when *R*_C_ = 30%.

For the armchair BP devices shown in Figure 4b, an obvious difference can be observed in that *G* decreases instantly when *R*_C_ *<* 15%, especially for the structures with longer pressure regions. For example, the conductance decreases by six orders of magnitude when *R*_C_ increases from zero to 15% for the device with a pressure region equal to 24*L*. By comparing Figure 4a and Figure 4b we found that the armchair BP device can be used as a pressure sensor, while the zigzag BP device cannot be used in that way when the pressure ratio is less than 15%. This is the core conclusion of this work.

To examine the influence of structural relaxation, the conductance of two fully relaxed zigzag and armchair BP devices with a length equal to 24*L* were also plotted as dashed curves with stars in Figure 4a and Figure 4b, respectively. The conductance of the fully relaxed BP devices is qualitatively in accordance with that of partially relaxed BP devices, except for the values are larger for the former. More importantly, the conductance is not sensitive to the pressure when *R*_C_ *<* 15% for the fully relaxed zigzag BP device, while it decreases rapidly for the fully relaxed armchair BP device.

### Band alignment analysis and WKB fitting

**Figure 5:**
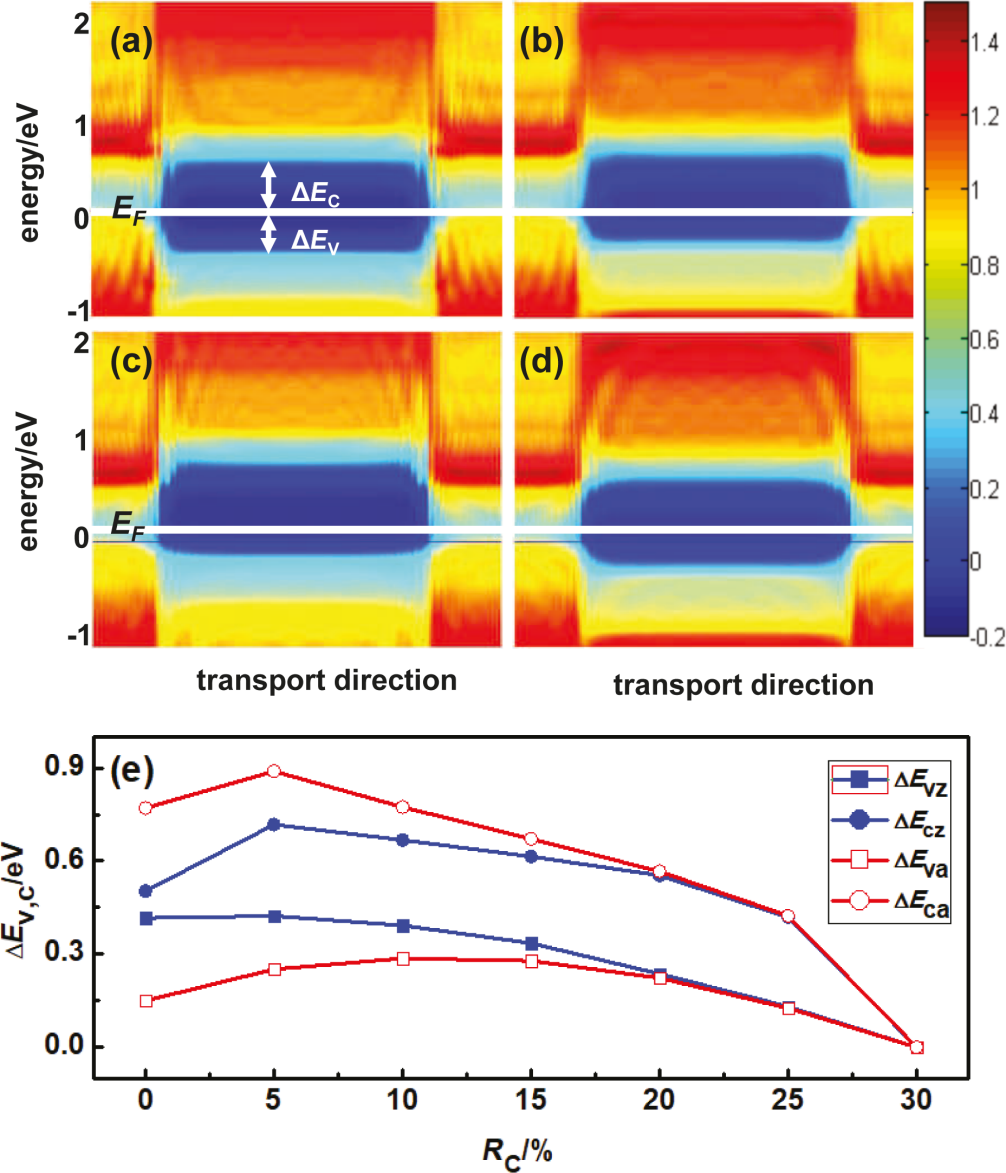
Real-space distribution of PDOS at the Fermi level in logarithmic scale along the transport direction for pure monolayer zigzag and armchair BP devices with a length of the pressure region equal to 24*L* and different values of *R*_C_. (a) Zigzag BP device with *R*_C_ = 0; (b) zigzag BP device with *R*_C_ = 15%; (c) armchair BP device with *R*_C_ = 0; (d) armchair BP device with *R*_C_ = 15%. In each panel, different colors describe the magnitudes of PDOS as indicated by the color bar, and the white horizontal line represents the Fermi level. (e) Δ*E*_V_ and Δ*E*_C_ as functions of *R*_C_ for zigzag (*E*_cz_ and *E*_vz_) and armchair (*E*_ca_ and *E*_va_) BP devices.

The different behavior of conductance as a function of the pressure for zigzag and armchair BP devices can be understood by analyzing the band alignment of the system. This can be shown intuitively by plotting the partial density of states (PDOS) in the scattering region along the transport direction. Figure 5a and Figure 5b show the PDOS at the Fermi level of zigzag BP devices with a length of the pressure region equal to 24*L* and *R*_C_ equal to 0 and 15%, respectively. When *R*_C_ = 0, the Fermi level of both leads locates in the center of conduction-band maximum (CBM) and valence-band maximum (VBM). When *R*_C_ reaches 15%, both VBM and CBM are pushed towards positive energy hinting to a p-type transport in the zigzag BP device. Figure 5c and Figure 5d show the PDOS at the Fermi level of armchair BP devices under the same conditions. When *R*_C_ = 0%, *E*_F_ is closer to the VBM. When *R*_C_ = 15%, both VBM and CBM are pushed towards negative energy, which is very different from the results obtained for the zigzag BP device.

To see more clearly the variation of VBM and CBM as functions of pressure, we also plotted the band offsets Δ*E*_V_ and Δ*E*_C_ as functions of *R*_C_ for zigzag devices and armchair devices with 24*L* pressure length in Figure 5e, where Δ*E*_V_ = *E*_F_ − VBM and Δ*E*_C_ = CBM − *E*_F_. For both zigzag and armchair BP devices, Δ*E*_V_ is always smaller than Δ*E*_C_ indicating the valence band domination of quantum transport for all BP devices, especially for the armchair BP devices. More interestingly, Δ*E*_V_ of the armchair BP devices increases as function of *R*_C_ when *R*_C_ is less than 15%, which explains the continuous decrease of conductance versus *R*_C_. While for the zigzag BP devices, the simultaneous decrease of Δ*E*_C_ and Δ*E*_C_ induces a roughly invariant conductance when *R*_C_ is less than 15%. When *R*_C_ is equal to 30%, Δ*E*_V_ and Δ*E*_C_ are zero because a semiconductor–metal phase transition occurs for the central BP section. Band-alignment analysis reveals the information of bandgap variation as shown in Figure 3d, and gives insight in the different conductance behavior between zigzag and armchair BP devices under finite pressure.

In view of the limitation of structure size in the first principles calculation, we performed an empirical WKB fitting with parameters obtained from the DFT calculations to predict the pressure-dependent conductance of pure BP devices at larger scales. Using the WKB method, the conductance *G*_WKB_ can be estimated empirically by [46–50]

[2] $$\ln G_{\text{WKB}}=-A\frac{l}{\sqrt{\frac{1}{m_{\text{C}}\Delta E_{\text{C}}}+\frac{1}{m_{\text{V}}\Delta E_{\text{V}}}}}+B,$$

where *A* and *B* are two parameters to be fitted; *l* is the length of the pressure region in the BP device; *m*_V_ and *m*_C_ are the effective masses of the electrons in the compressed monolayer BP at VBM and CBM, respectively, which were obtained using the deformation potential theory based on the first principles results [3]. For a given BP device, *A* and *B* can be obtained from Equation (Equation 2). The evaluated *G*_WKB_ for pure zigzag and armchair BP devices are also shown in Figure 4a and Figure 4b, respectively, by the open symbols to examine the accuracy of WKB modeling. Obviously, *G*_WKB_ is quantitatively accordant with *G* from the first principles calculation indicating the reliability of the WKB calculation.

**Figure 6:**
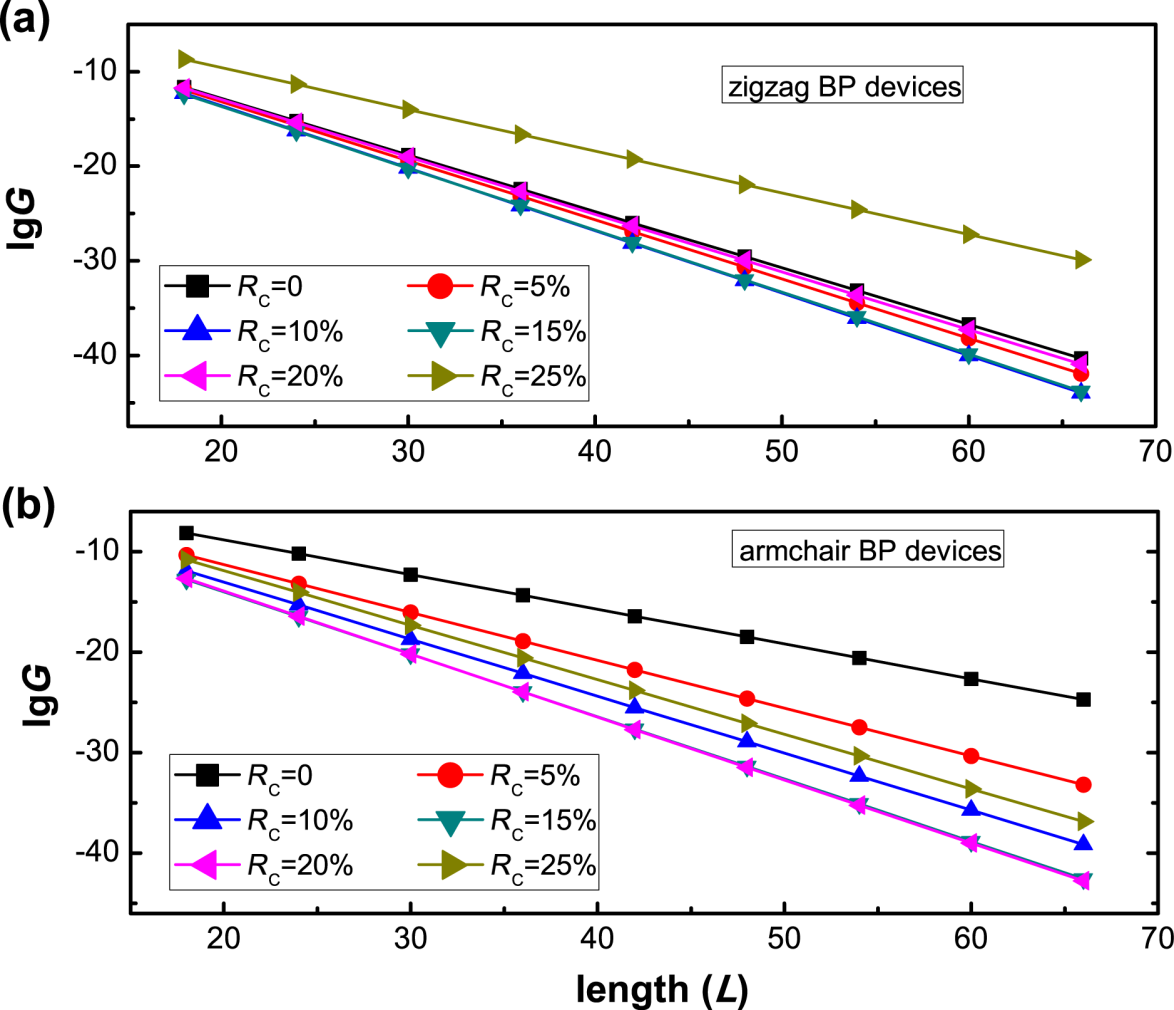
The values of ln(*G*_WKB_) as function of the length of the pressure region for (a) zigzag and (b) armchair BP devices at different values of *R*_C_.

Furthermore, we employed the fitted parameters *A* and *B* to predict the pressure-dependent conductance of zigzag and armchair BP devices with arbitrary lengths. Figure 6a and Figure 6b show ln(*G*_WKB_) as a function of the length of the pressure region *L* for zigzag and armchair BP devices at different values of *R*_C_, respectively. The parameters *A* and *B* are listed in Table 1. For both zigzag and armchair BP devices, the pressure-dependent conductance decreases linearly with the length of the structures. The exponential decay of conductance versus length of structure is reasonable because the resistance is mainly dominated by the semi-conducting BP section in the scattering region of the pure BP devices.

**Table 1:** Fitted parameters *A*’ = *−A*/[(1/*m*_C_Δ*E*_C_) + (1/*m*_V_Δ*E*_V_)]^0.5^ and *B* for different values of *R*_C_. *A*’_z_ and *B*_z_ are for zigzag BP devices, and *A*’_a_ and *B*_a_ are for armchair BP devices.

*R*_C_	*A*’_z_	*B*_z_	*A*’_a_	*B*_a_
				
0%	0.5975	−0.8752	0.3458	−1.8954
5%	0.6250	−0.6674	0.4758	−1.7785
10%	0.6605	−0.3853	0.5675	−1.6757
15%	0.6547	−0.5780	0.6212	−1.5694
20%	0.6085	−0.7531	0.6264	−1.4071
25%	0.4416	−0.7330	0.5426	−1.0385

## Conclusion

We investigated the pressure-dependent quantum transport properties of pure zigzag and armchair BP devices using first principles calculations. When the pressure ratio is smaller than 15%, the conductance of zigzag BP devices changes very little with pressure, while the conductance of armchair BP devices decreases distinctly with large magnitude. That means the armchair BP devices can work as pressure sensors, but the zigzag BP devices cannot. The sensitivity is proportional to the length of the pressure region in the device. Band alignment was analyzed to help understand the physics of this behavior. In view of the constraints of first principles calculation regarding the device size, we fitted a set of parameters using the WKB method based on the first principles results to predict the pressure-dependent conductance of BP devices of arbitrary length.

## Acknowledgements

This work was financially supported by grants from the National Natural Science Foundation of China (Grant No. 11774217, 11774238, 11774239, 11704190 and 11404273), Shenzhen Key Lab Fund (ZDSYS20170228105421966), the Jiangsu Provincial Natural Science Foundation of China (Grant No. BK20171030). X.M. Rong and Z.W. Wu were partially supported by the postgraduate research opportunities program of Hongzhiwei Technology (Shanghai) Co., Ltd. (hzwtech-PROP).
